# Innovating and expanding weight loss strategies for breast cancer survivors

**DOI:** 10.18632/oncotarget.27898

**Published:** 2021-03-16

**Authors:** Jennifer Y. Sheng, Vered Stearns

**Keywords:** breast cancer, obesity, lifestyle interventions

***Comment on:** The Effects of a Remote-based Weight Loss Program on Adipocytokines, Metabolic Markers, and Telomere Length in Breast Cancer Survivors: the POWER-Remote Trial. Clin Cancer Res. 2020; 26:3024–3034. https://doi.org/10.1158/1078-0432.CCR-19-2935. [PubMed]*

**Figure 1:**
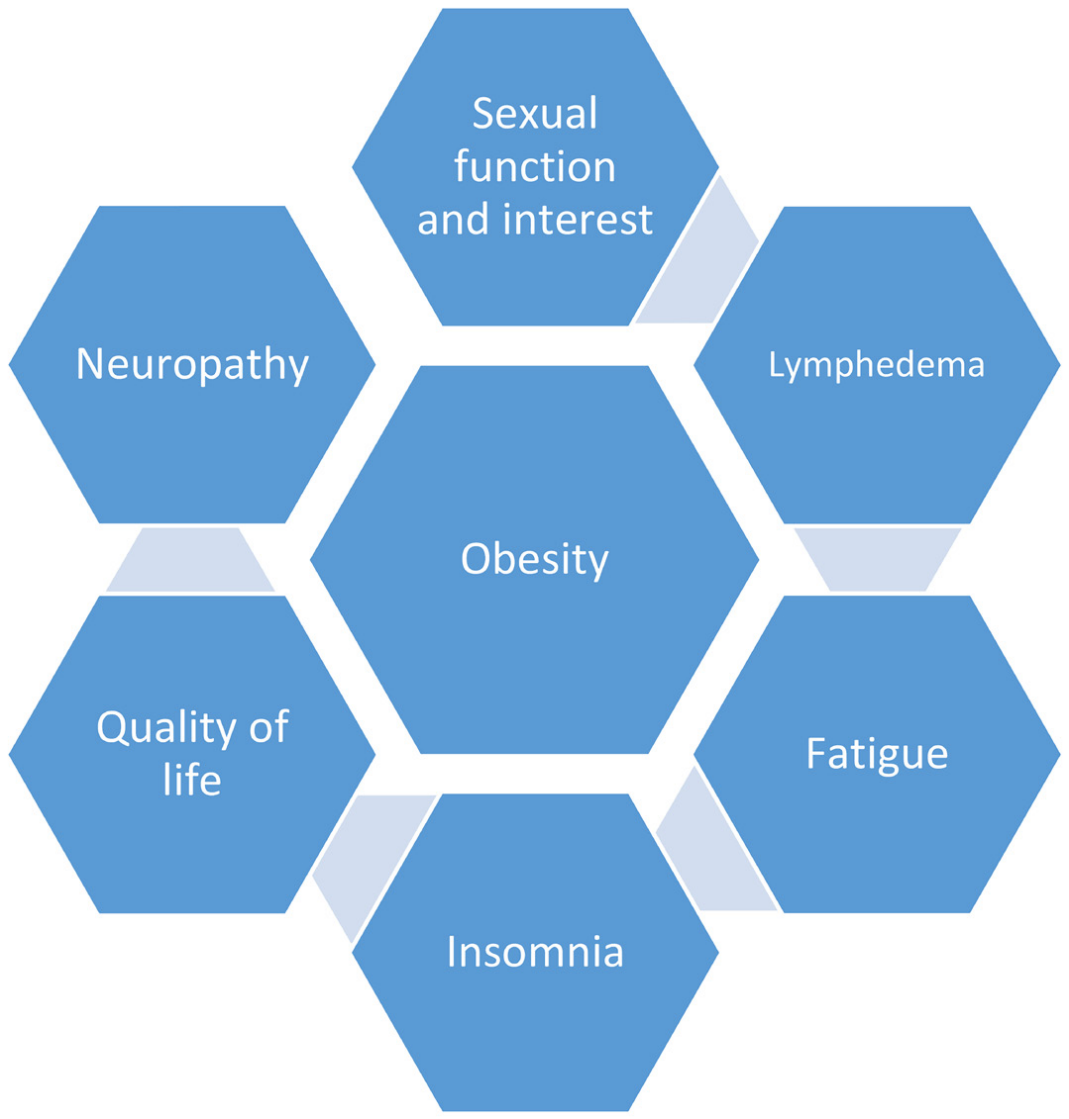
The impact of obesity at and following a breast cancer diagnosis.

Overweight and obesity are prevalent in over two thirds of the general population in the United States and are associated with an increased risk of malignancies, including breast cancer [1]. Up to 96% of women gain weight following a diagnosis of breast cancer [2]. This weight gain may increase risk of recurrence by 40–50%, and breast cancer-related mortality by 53–60% [3–5]. Compared to those with ideal body weight, women who have excess weight experience inferior outcomes once diagnosed with breast cancer, despite standard local and adjuvant therapy [6]. Obesity at and following a breast cancer diagnosis is associated with poor quality of life and increased risk of adverse treatment effects (Refer to (Figure 1)) [7–19]. Clinically significant weight loss of ≥ 5% is associated with decreased all-cause mortality and prevalence of prediabetes and cardiovascular disease [20, 21]. Thus, survivorship guidelines recommend that weight loss should be a priority for overweight/obese survivors [22].

Numerous clinical trials have tested behavioral strategies for weight loss in survivors of early stage breast cancer, including modifications in diet, remote or group interventions [23]. The original Practice-based Opportunities for Weight Reduction (POWER) study in obese individuals with a risk for cardiovascular disease demonstrated equivalent weight loss outcomes between in-person coaching and a remote intervention [24]. Our group compared the remote-based POWER intervention (telephone calls by a coach, access to online learning materials, online self-directed dietary/activity monitoring) to self-directed weight loss in overweight or obese survivors of early-stage breast cancer [25]. We evaluated several outcomes including: (i) whether a 12-month remotely delivered behavioral weight loss intervention (POWER-remote) allows a greater proportion of breast cancer survivors to achieve ≥ 5% weight compared to a self-directed approach, (ii) modulation of biomarkers of cancer risk including metabolism, inflammation, and telomere length, and (iii) changes in patient-reported outcomes (PROs) [26].

At 6 months, 51% of women randomized to POWER-remote lost ≥ 5% of their baseline body weight, compared with 12% in the self-directed arm; proportions were similar at 12 months (51% vs 17%). Additionally, weight loss correlated with significant decreases in leptin, and favorable modulation of inflammatory cytokines and lipid profiles. While levels of inflammatory cytokines improved, changes were not statistically significant. However, studies show that weight loss of ≥ 10% has been associated with modulation of serum and tissue biomarkers, such as Ki-67, adiponectin, adiponectin to leptin ratio, sex hormone binding globulin, estradiol, testosterone and insulin [27]. Thus, greater weight loss may be necessary to reverse some of these deleterious biological changes associated with obesity. Larger data sets testing relevant biomarkers are necessary.

Our secondary analyses of PROs demonstrate that study arm did not influence improvement in PROs, but that weight loss of ≥ 5% of baseline weight was associated with significant improvement in physical function compared to those who lost < 5% of baseline weight (ASCO 2020). These analyses suggest that a remove behavioral intervention by itself may not be sufficient to improve PROs, and that weight loss may be the most critical to favorably influencing these. Previous studies demonstrate that weight loss of ≥ 10% has been associated with greater improve physical function as measured by physical performance test and functional status questionnaire [28].

Others have demonstrated that deficient sleep may directly contribute to obesity and undermine weight loss efforts via multiple pathways, including reduction in physical activity and increase in consumption of calorie dense foods [29–31]. Post hoc analysis revealed that participants receiving the POWER-remote intervention who reported poor sleep at baseline had a lower weight loss percentage than those with better sleep receiving the intervention (4.1% v 6.8%) (SABCS 2020). While not statistically significant, these findings are clinically meaningful and further studies are needed to determine if detection and treatment of sleep disturbances is a critical and neglected component of weight loss treatment. We have recently completed enrollment to a study designed to determine whether an intervention that detects and treats underlying sleep disturbance augments behavioral strategies for weight loss (NCT03542604).

Our intervention is feasible and scalable, given it incorporated telephone calls by a coach, accelerometers to assess activity, and self-directed dietary/activity monitoring. A few important features of the POWER-remote study set it apart from other weight loss studies in breast cancer survivors. First, it confirms that weight loss outcomes at 6 months parallel those at 12 months. This is critical to acknowledge as it allows greater efficiency in implementation through appropriate allocation of resources among interested individuals. Second, the impact of behavioral weight loss interventions on biomarkers suggests that lifestyle modifications have the potential to reduce risk of new or recurrent cancer, and other illnesses. Lastly, while weight loss may lead to improvements in physical function, benefits may be hampered by patient related factors, such as poor sleep.

Our data reinforce that a remotely delivered weight loss intervention can result in significant weight loss in over half of participants. However, our results coupled with other reported weight loss interventions in breast cancer survivors also highlight that current weight loss approaches yield limited results in almost half of participants [32, 33]. Therefore, there is a need to develop additional behavioral strategies or consider augmentation with pharmacological or other approaches. Understanding and designing novel interventions to improve other patient related factors such as physical function, mood and sleep, should also be considered. While it is clearly desirable to have a precision approach for individual patients, the task of building such models is challenging.

Studies in the general obese population indicate that initial weight loss response at 4–12 weeks predicts weight loss at 1 year and beyond, emphasizing the need for the addition of new strategies to behavioral interventions [34–37]. One approach that we developed in a prospective, non-randomized clinical trial includes an assessment of weight loss trajectory with a behavioral intervention followed by augmentation with anti-obesity pharmacotherapy for those who do not lose ≥ 5% of their weight at 8 weeks (NCT04499950). An anti-obesity agent may catalyze behavioral weight loss by improving dietary and physical activity behaviors.

As there may be barriers to in-person or group interventions and studies aim for scalability, we can expect remote interventions like POWER-remote to be used as a practical strategy for weight loss. Remote strategies that incorporate a comprehensive assessment and coaching are necessary to ensure safe and cost effective treatment for cancer survivors struggling with weight loss. Both patient and provider education regarding these approaches will also be important aspects of dissemination of knowledge and optimization of care delivery. Other challenges that remain include understanding modulators of obesity, such as biomarkers, microbiome, clinical data, sleep and PRO. While personalized approaches open the possibility to better predict benefits, they may also add complexity in cost and scale. Thus, addressing the payer structure, expanding coverage for obesity treatments, and delineating participants and payer incentives are critical for creating sustainable and accessible weight loss approaches. Meeting all these challenges will require collaboration among researchers, clinicians, statisticians, and social scientists.
